# Nursing Students' Personality Traits and Their Attitude toward Artificial Intelligence: A Multicenter Cross-Sectional Study

**DOI:** 10.1155/2024/6992824

**Published:** 2024-08-02

**Authors:** Gihan Mohamed Mohamed Salem, Heba Emad El-Gazar, Abeer Yahia Mahdy, Talal Ali F. Alharbi, Mohamed Ali Zoromba

**Affiliations:** ^1^ Psychiatric and Mental Health Nursing Department Faculty of Nursing Benha University, Banha, Egypt; ^2^ Department of Nursing College of Applied Medical Sciences University of Bisha, Bisha, Saudi Arabia; ^3^ Administration Department Faculty of Nursing Port Said University, Port Fuad, Egypt; ^4^ Medical-Surgical Nursing Department Faculty of Nursing Najran University, Najran, Saudi Arabia; ^5^ Medical Surgical Nursing Department, Faculty of Nursing Benha University, Benha, Egypt; ^6^ Department of Community Psychiatric and Mental Health Nursing College of Nursing Qassim University, Buraidah, Saudi Arabia; ^7^ College of Nursing Sulaiman Al Rajhi University, Al Bukayriah, Saudi Arabia; ^8^ Nursing Department College of Nursing Prince Sattam Bin Abdulaziz University, Al-Kharj, Saudi Arabia; ^9^ Department of Psychiatric and Mental Health Nursing Faculty of Nursing Mansoura University, Mansoura, Egypt

## Abstract

**Background:**

Despite the importance of studying factors contributing to nursing students' attitudes toward artificial intelligence, yet according to our knowledge, no study has addressed the relationship between personality traits and the attitude of nursing students toward artificial intelligence.

**Aim:**

This study aimed to unveil whether nursing students' personality traits are related to their attitude toward AI.

**Methods:**

This multicenter cross-sectional study included 218 nursing students from three governmental universities across various regions of the Kingdom of Saudi Arabia. Data were gathered online, utilizing the Big Five Inventory, the General Attitudes toward Artificial Intelligence Scale, and a demographic questionnaire. Descriptive statistics, Pearson's correlation, and regression analysis were employed. The research complied with the STROBE checklist.

**Results:**

Findings indicated that nursing students with a high score in the openness trait displayed positive attitudes toward artificial intelligence. Conversely, those who scored high in neuroticism and agreeableness exhibited fewer positive attitudes toward artificial intelligence and more negative attitudes toward artificial intelligence. Additionally, nursing students who ranked high in conscientiousness showed a negative attitude toward artificial intelligence.

**Conclusion:**

Except for extraversion, personality traits appear to predict attitudes toward artificial intelligence. *Implications for Nursing Management*. The current study provides a foundation for understanding how generative AI can be integrated into nursing education and practice in a manner that is both effective and considerate of the diverse psychological profiles of students.

## 1. Introduction

Artificial intelligence (AI) is progressively shaping the scene of healthcare, with its integration becoming more pervasive across various domains such as patient care, diagnostics, and administrative tasks [1]. In recent years, the healthcare sector has perceived a surge in the adoption of AI technologies, ranging from machine learning algorithms aiding in disease prediction to robotic assistance in surgical procedures [2]. The potential of AI to enhance efficiency, accuracy, and patient outcomes is widely acknowledged, necessitating a workforce that is adept and comfortable working alongside these technologies [3].

The integration of AI in healthcare settings is challenging due to trust, ethics, and job security concerns [4]. Addressing these issues proactively and fostering a positive outlook toward AI among nursing students can pave the way for smoother integration and optimal utilization of AI in healthcare [5]. Understanding nursing students' attitudes toward AI is crucial as it influences their future interactions with AI technologies and integration into healthcare practices [6]. Attitudes can vary, with younger, tech-savvy students often embracing AI, while older or less tech-savvy individuals may be skeptical [7, 8]. As future healthcare professionals, nursing students' attitudes will shape the broader workforce's readiness for AI [9, 10]. Studying these attitudes provides insights into the educational needs and interventions required to prepare future nurses for the AI-driven healthcare landscape. There is a pressing need to incorporate AI-related content into nursing curricula and training programs to equip students with the necessary knowledge and skills [11, 12]. By exploring the factors influencing these attitudes, educators and policymakers can devise targeted strategies to foster positive attitudes and readiness for AI integration [9].

The relationship between personality traits and attitudes toward technology or innovation has been the subject of extensive research, with several studies highlighting the significance of individual personality characteristics in shaping one's perception and acceptance of technology [13]. Drawing upon the technology acceptance model, which postulates that perceived affluence of usage and perceived helpfulness are key determinants of technology acceptance, it can be inferred that personality traits play a critical role in shaping these perceptions [14]. Moreover, the Big Five personality traits model provides a comprehensive framework to understand how different personality dimensions may influence attitudes toward AI [15, 16].

The Big Five personality traits framework categorizes personality into five dimensions: openness, conscientiousness, extraversion, agreeableness, and neuroticism (OCEAN). Openness involves receptiveness to new experiences; conscientiousness denotes diligence and responsibility; extraversion encompasses sociability; agreeableness signifies compassion and cooperation; and neuroticism involves emotional instability and anxiety [17]. Individuals with high openness are likely to have positive attitudes toward AI due to their receptiveness to change [18]. In contrast, those high in neuroticism may have negative attitudes toward AI, perceiving it as threatening. Highly agreeable individuals might also be more skeptical of AI's impact on interpersonal aspects of patient care [19].

In the context of AI in healthcare, it is plausible to expect that these personality traits would play a significant part in shaping nursing students' attitudes toward AI. For instance, students high in conscientiousness may exhibit meticulousness in their interactions with AI, ensuring that all protocols are followed, but they may also be more critical of AI's potential limitations. Extraversion may influence how nursing students communicate and collaborate with AI technologies, with more extroverted students potentially being more open to engaging with AI in team-based settings. Thus, understanding how each of these personality traits correlates with attitudes toward AI is crucial for developing targeted educational and training interventions [11, 12].

Despite the increasing interest in the impact of AI on healthcare and the importance of fostering positive attitudes toward AI among healthcare professionals, there is a noticeable gap in the literature concerning the specific relationship between nursing students' personality traits and their attitudes toward AI [9]. Most existing studies have focused on healthcare professionals' attitudes toward AI in general, without delving into the nuanced ways in which individual personality traits may influence these attitudes [20–23].

Recent studies have begun to explore the intersection of AI, healthcare, and personality traits in various cultural settings. For example, research in South America has examined the role of personality in the acceptance of AI among healthcare workers, revealing distinct patterns influenced by cultural values and healthcare systems [18]. Similarly, studies from Africa have shed light on the unique challenges and opportunities presented by AI in healthcare, emphasizing the need for culturally sensitive approaches to AI integration [24]. In Asia, the rapid adoption of AI in healthcare has prompted investigations into the influence of personality traits on AI acceptance among nursing students and professionals, highlighting differences in attitudes compared to Western counterparts [9, 25, 26].

Furthermore, the few studies that have explored the relationship between personality traits and attitudes toward technology in healthcare have largely been conducted in Western and Eastern contexts, leaving a gap in our understanding of how these dynamics play out in the Middle East, especially the Kingdom of Saudi Arabia. Addressing this gap is imperative, as the cultural and educational context of the Kingdom of Saudi Arabia presents unique challenges and opportunities in the integration of AI in healthcare. The country has been making significant strides in incorporating AI into various sectors, including healthcare, as part of its Vision 2030 Strategic Plan [27–29]. However, to fully realize the potential of AI in enhancing healthcare delivery, it is crucial to understand how nursing students, who represent the future of the healthcare workforce in the region, perceive and interact with AI.

*Aim of the Study*. This study aimed to investigate the relationship between nursing students' personality traits and their attitudes toward AI.

### 1.1. Objectives

1. To investigate the relationship between nursing students' personality traits and their attitudes toward artificial intelligence (AI)
2. To identify the distinct personality traits of nursing students who have positive attitudes toward AI compared to those with negative attitudes
3. To determine which specific personality traits (e.g., openness, conscientiousness, extraversion, agreeableness, and neuroticism) predict nursing students' attitudes toward AI

### 1.2. Hypotheses

1. H1: nursing students' personality traits are significantly associated with their attitudes toward AI
2. H2: nursing students with a positive attitude toward AI exhibit distinct personality traits compared to those with a negative attitude toward AI
3. H3: specific personality traits (e.g., openness, conscientiousness, extraversion, agreeableness, and neuroticism) predict nursing students' attitudes toward AI

## 2. Methods

### 2.1. Design

A multicenter cross-sectional online survey was performed.

### 2.2. Participants and Setting

We recruited nursing students using convenience sampling from three governmental universities across three regions of the Kingdom of Saudi Arabia: Prince Sattam Bin Abdulaziz University, Bisha University, and Najran University. Eligible participants were undergraduate nursing students, from any year of their bachelor's program, at one of the three specified governmental universities in the Kingdom of Saudi Arabia, and willing to engage in the survey. Exclusion criteria include postgraduate students, students with mental health challenges or undergoing mental health therapy, or those enrolled in self-improvement or professional development courses.

Calculations for determining the sample size were conducted using G^*∗*^Power software (version 3.1.9.4), leveraging a linear model calculation method [30]. The parameters employed consisted of a power level of 0.95, an alpha level of 0.01, an effect size of *d* = 0.15 [31], and 9 predictors (five related to personality traits and four to demographics). These parameters pointed to a need for a minimum of 214 participants. A total of 218 nursing students completed the online survey.

### 2.3. Measures

The Big Five Inventory (BFI) and the General Attitudes toward Artificial Intelligence Scale (GAAIS), advanced in English, and a demographic questionnaire were used in the current study. Together, the English scales were translated into Arabic using the translation-back-translation technique [32]. Initially, the English scale was translated into Arabic by the primary researcher, and a skilled translator subsequently performed a back translation from Arabic to English. The back-translated version was then cross-checked with the original English scale, resulting in certain corrections to ensure a close alignment with the original content. A team of five nursing professors, who are experts in the field, revised the translated form in comparison with the genuine one to confirm that the terms used were equivalent and that the language was clear and easy to understand. After translation, the scales were initially tested with 31 nursing students to confirm the validity of the final version. Both scales used a 5-point Likert scale for responses, with the scoring system ranging from 1 (strongly disagree) to 5 (strongly agree).

#### 2.3.1. BFI

This scale was established by Benet-Martinez and John [33] to assess the five major personality traits using 44 items. These traits are “extraversion (8 items), agreeableness (9 items), conscientiousness (9 items), neuroticism (8 items), and openness (10 items).” Among nursing students, the trait with the highest score reflects their most dominant personality characteristic. In its original form, Cronbach's alpha for the scale varied from 0.65 to 0.84. In the current study, a confirmatory factor analysis (CFA) was conducted, contrasting two models. The initial model grouped all items of scale under one factor, whereas the later model examined a suggested structure of five distinct factors. The data suggested that the five-factor model was more fitting than the single-factor model, as evidenced by the following metrics: *χ*^2^ = 1453.043, df = 892, *χ*^2^/df = 1.629, CFI = 0.90, IFI = 0.89, and RMSEA = 0.054, in contrast to *χ*^2^ = 4964.743, df = 911, *χ*^2^/df = 5.450, CFI = 0.21, TLI = 0.21, and RMSEA = 0.143 for the single-factor model.

#### 2.3.2. GAAIS

This scale was established by Schepman and Rodway [34] to gauge people's general attitudes toward AI. This scale encompasses 20 items with two dimensions: positive attitude toward AI (12 items) and negative attitude toward AI (8 items). Each dimension yields a separate score, with higher scores reflecting more intense attitudes, be they positive or negative, toward AI. In the original study, Cronbach's alpha for the positive dimension was 0.88 and 0.83 for the negative dimension. In this study, the CFA indicated that the two-factor model exhibited a more satisfactory structure compared to a one-factor model (*χ*^2^ = 281.195, df = 169, *χ*^2^/df = 1.664, IFI = 0.96, CFI = 0.96, and RMSEA = 0.055 vs. *χ*^2^ = 619.023, df = 170, *χ*^2^/df = 3.641, TLI = 0.82, CFI = 0.82, and RMSEA = 0.110).

#### 2.3.3. The Demographic Questionnaire

The demographic questionnaire involved data about age, gender, marital status, and educational level.

### 2.4. Data Collection and Ethical Consideration

Data were collected through an online survey created using Google Forms, which is a readily available web-based survey platform. The survey link, attended by invitation, was disseminated to students via email and through private student Telegram groups affiliated with each university. The invitation letter outlined the study's purpose and specified the target participants. Participation was entirely voluntary, and confidentiality was guaranteed. An online informed consent method was provided, and participants were required to acknowledge their agreement by clicking on an “I agree” button before proceeding to the survey. To ensure data quality, all survey questions were mandatory, preventing the submission of incomplete responses. Additionally, measures were in place to prevent participants from submitting multiple responses using a similar account or device. Once the number of submissions surpassed the predetermined sample size, data collection was halted. The data collection period spanned from mid-March to mid-June 2023. The Standing Committee of Bioethics Research (SCBR), Prince Sattam Bin Abdulaziz University, approved the study (Approval No. SCBR-181/2023).

### 2.5. Statistical Analysis

Statistical analyses were performed using SPSS version 27.0, with validity analysis performed in Amos version 23.0. Descriptive statistics, including counts, percentages, means, and standard deviations, were performed to depict participants' demographic features and study variables. Measures were taken to ensure the validity and reliability of the study variables. Differences in study outcomes based on demographic characteristics were examined using independent-samples *t*-tests and one-way analyses of variance (ANOVA). The Pearson *r* correlation coefficient was applied to ascertain the relationships between personality traits and attitudes toward AI. Stepwise multiple regression analyses were carried out for both positive and negative attitudes toward AI, focusing on variables that showed significance in the prior difference and correlation tests. To ensure model stability and the absence of multicollinearity, both tolerance and variance inflation factor metrics were examined and confirmed to be within acceptable limits.

### 2.6. Common Method Bias (CMB)

The data for the current research were obtained solely from a lone source via self-reported methods, thereby heightening the risk of CMB. To mitigate current potentiality, both statistical and procedural strategies were implemented. Procedurally, participants received a thorough orientation regarding the study's aims, and assurances of voluntary participation, unwavering anonymity, and strict confidentiality were provided. Furthermore, the items within the study's scales were randomized and subjected to preliminary testing to augment clarity [35]. On the statistical front, Harman's single-factor test was adopted [36]. The results indicated that only 18.47% of the variance was due to a single factor, which is well below the 50% threshold [37], suggesting that CMV was not a predominant concern. Furthermore, the CFA of the scales addressing personality traits and attitudes toward AI demonstrated a satisfactory fit with a multifactor model than with a singular-factor model, pointing to an absence of widespread CMV.

## 3. Results

### 3.1. Preliminary Analysis

The study participants were predominantly female (81.7%), unmarried (88.1%), and were in their fourth year of education. The majority of the participants were aged 20 years or older, constituting 55.5%. There was no statistically significant difference in attitudes, positive or negative, toward AI based on the demographics of the nursing students. However, there were specific differences observed across the educational levels of students, and the mean positive attitude score was highest among second-year students (*M* = 3.62), while the mean negative attitude score was highest among third-year students (*M* = 2.61). Interns had the lowest mean positive attitude score (*M* = 3.22), and first-year students had the lowest mean negative attitude score (*M* = 2.21) (Table 1).

### 3.2. Reliability and Validity

A CFA was conducted to validate the study scales. Initially, the Kaiser–Meyer–Olkin (KMO) measure and the Bartlett test of sphericity assessed the suitability of the sample. A KMO measure should ideally exceed 0.60, while the Bartlett test of sphericity ought to be statistically significant at *P* < 0.05. The findings showcased KMO values of 0.861 (*P* < 0.001) for the scale assessing personality traits and 0.953 (*P* < 0.001) for the scale measuring attitudes toward AI. Factor loadings for all scale items surpassed the benchmark of 0.5 [38], corroborating the scales' construct validity. In addition, all average variance extracted (AVE) exceeded the 0.50 benchmark for the study variables, signifying adherence to convergent validity. The AVE values were observed to be greater than the maximum shared variance (MSV), thus reinforcing the discriminant validity of the research model [39] (Table 2).

Reliability was further scrutinized using Cronbach's alphas and composite reliability (CR). Study results revealed Cronbach's alpha values spanning from 0.89 to 0.92 for all scale factors, exceeding the 0.7 threshold [40], implying robust internal consistency. Additionally, CR values ranged between 0.89 and 0.93 for all study scale factors, all surpassing the 0.70 threshold [41], underscoring the scales' elevated reliability (Table 2).

### 3.3. Descriptive Statistics

As presented in Table 2, the personality traits of the nursing students who contributed in this study ranked as follows: “conscientiousness” (*M* = 3.18, SD = 0.75), “openness” (*M* = 3.10, SD = 0.75), “agreeableness” (*M* = 2.90, SD = 0.77), “extraversion” (*M* = 2.67, SD = 0.81), and “neuroticism” (*M* = 2.18, SD = 0.68). The participating nursing students exhibited a more positive attitude toward AI (*M* = 3.37, SD = 0.81) compared to a negative attitude toward AI (*M* = 2.39, SD = 0.79).

### 3.4. Correlations

Table 3 presents the results of the Pearson correlation analysis. It indicates that the positive attitude toward AI significantly and positively correlates with the openness personality trait (*r* = 0.499, *P* < 0.001) and negatively correlates with both the agreeableness (*r* = −0.153, *P*=0.024) and neuroticism (*r* = −0.142, *P*=0.036) personality traits. Additionally, the results revealed a significant positive correlation between negative attitude toward AI and agreeableness (*r* = 0.488, *P* < 0.001), conscientiousness (*r* = 0.316, *P* < 0.001), and neuroticism (*r* = 0.319, *P* < 0.001) personality traits.

### 3.5. Stepwise Multiple Regression Analysis of Attitude toward AI

A stepwise multiple regression investigation was done to explore how nursing students' attitudes toward AI, either positive or negative, as the dependent variable, correlated with personality traits that showed statistical significance in Pearson's correlation analysis as independent variables. Sociodemographic factors were not included in the regression model since they did not show any significant impact on the attitudes toward AI (as detailed in Table 1). The analysis found no multicollinearity issues; for positive attitudes, the tolerance values were between 0.86 and 0.97 (above 0.1), and the variance inflation factors (VIFs) were between 1.04 and 1.17 (below 3). Similarly, for negative attitudes, tolerance values ranged from 0.78 to 0.89 (above 0.1), and VIF values ranged from 1.12 to 1.28 (below 3).

In the regression analysis predicting positive attitudes toward AI, the results indicated that high openness (*β* = 0.499, *P* < 0.001), low neuroticism (*β* = −0.178, *P*=0.005), and low agreeableness traits (*β* = −0.175, *P*=0.019) were significant predictors of positive attitudes toward AI. The model accounted for 27.2% of the variance in nursing students' attitudes toward AI (Table 4).

In the regression analysis predicting negative attitudes toward AI, the results displayed that agreeableness (*β* = 0.372, *P* < 0.001), conscientiousness (*β* = 0.172, *P*=0.005), and neuroticism (*β* = 0.160, *P*=0.011) were significant predictors of negative attitudes toward AI. The model accounted for 28.7% of the variance in nursing students' negative attitudes toward AI (Table 5).

## 4. Discussion

This study examined the relationship between nursing students' personality traits and their attitudes toward artificial intelligence (AI), focusing specifically on a Saudi Arabian context. While there have been studies examining healthcare professionals' attitudes toward AI, limited research has focused on nursing students in Saudi Arabia. This study aimed to fill that gap by exploring how personality traits, as categorized by the Big Five framework, influence attitudes toward AI.

The study findings indicated no statistically significant difference in the students' attitudes toward AI, either positive or negative, while taking into account the demographic characteristics of the students. Previous studies have suggested that cultural factors can influence attitudes toward technology, supporting the idea that a similar cultural background may lead to consistent attitudes [11, 18, 20]. This lack of difference could be attributed to the homogeneous cultural background of the participants. Saudi Arabian nursing students may share similar cultural values and educational experiences, which could result in comparable attitudes toward AI. However, there is limited direct evidence linking similar cultures as predictors of attitudes toward AI. Other possible explanatory factors include varying levels of exposure to AI technologies and differences in educational curricula, which can significantly impact attitudes. Further research in diverse cultural settings is needed to fully understand the cross-cultural differences in attitudes toward AI.

### 4.1. Personality Traits and Attitudes toward AI

Overall, the findings conclude that the Big Five personality traits—openness to new experiences, agreeableness, neuroticism, and conscientiousness—all tend to predict attitudes toward artificial intelligence, whether they are positive or negative, with the exception of extraversion: nursing students who scored highly on the openness trait had positive attitude toward AI; in contrast, those with high neuroticism and agreeableness scores showed more negative than positive attitudes toward AI; moreover, conscientiousness-scoring nursing students had negative attitudes toward artificial intelligence. Thus, the findings of the study align with recent studies that found personality traits, to typically have a substantial effect on people's attitudes toward AI [8, 15, 18, 19, 42].

#### 4.1.1. Openness

The findings are in line with other authors who contend that people who score highly on openness to experience are more likely to be flexible, curious, respectful of innovation, and open to new technologies—all of which are indicators of positive attitudes toward artificial intelligence. This supports existing knowledge that openness is associated with positive perceptions of technology [13, 19].

#### 4.1.2. Agreeableness

However, the results of our study are consistent with suggestions that agreeableness was a significant predictor of people's negative attitudes toward AI [13, 43, 44]. Likewise, recent studies discovered no significant relationship between agreeableness and positive attitudes toward AI [19, 44]. In an explanation of such results, studies argued that people with higher agreeableness scores—such as etiquette, social skills, and willingness to compromise nature—might be more able to withstand the negative effects of AI [13, 43–45].

#### 4.1.3. Conscientiousness

In the current study, nursing students who ranked high in conscientiousness showed a negative attitude toward artificial intelligence. However, a research study stated that consciousness did not significantly predict positive or negative attitudes toward AI; nevertheless, it showed a weak but significant positive correlation with negative attitudes [13, 46, 47]. However, those who are highly conscientious may have a more positive opinion of AI since they place great importance on organization, efficiency, and observing regulations. These individuals may also see AI as a tool to boost efficiency and production as they understand the importance of aptitude, efficacy, and organization [19]. This highlights a mixed understanding of how conscientiousness influences AI attitudes, suggesting further investigation is needed.

#### 4.1.4. Neuroticism

The findings of our study showed that those who scored high in neuroticism exhibited more negative than positive attitudes toward artificial intelligence. Similarly, another study indicated that those who score highly on the neuroticism trait are more likely to be skeptical, fearful, and anxious, about the potential risks and implications of AI and consequently have negative attitudes toward artificial intelligence [19]. They may see AI as a threat rather than a helpful tool. The idea of machines mimicking human capabilities and potentially taking over tasks that were traditionally performed by humans may trigger feelings of insecurity and unease for these individuals. Their fear of AI replacing human jobs and potentially causing unemployment can further contribute to their negative attitudes [42, 48].

#### 4.1.5. Extraversion

Although extraversion is a personality characteristic that may predict attitudes toward AI generally [44], our study indicates that extraversion is not a significant predictor of favorable or negative attitudes toward AI for Saudi Arabian nursing students involved in our sample. These results seem to be in line with studies that showed extroversion not to be a significant predictor of attitudes toward artificial intelligence, either positive or negative [13, 19, 46]. One possible explanation is that students come from a cultural background where solidarity and social harmony are valued more highly than individuality. This might lead to less extraversion and a more cautious approach toward artificial intelligence [49]. This may suggest that extraversion may not be a strong predictor of attitudes toward artificial intelligence in this specific population. Conversely, Schepman [44] showed a negative correlation between extroversion and attitudes toward AI, suggesting that introversion traits increase positive attitudes toward AI [44].

One possible explanation of the results of our study could be the cultural context in Saudi Arabia. In cultures where collectivism and conformity are emphasized, individual personality traits such as extraversion may have a diminished influence on attitudes toward technology. However, because artificial intelligence is still an evolving and complex area, nursing students may have varying degrees of exposure to and awareness of its potential benefits and implications for their field of work.

### 4.2. Implications of the Study

Given the paucity of research on the personality traits and attitudes of Saudi Arabian nursing students toward artificial intelligence, the current study is crucial for examining the attitudes of these students and their preparedness to adopt AI in the field of nursing. Understanding the viewpoints and experiences of nursing students is imperative to address the scarcity of nurses, advance nursing, and improve patient outcomes.

Focusing on Saudi Arabia provides valuable insights into how cultural and educational contexts influence the acceptance of AI among future nurses. This information can help tailor AI healthcare systems to better align with the preferences and mindsets of their users, leading to more effective and efficient integration of AI in nursing practices. By doing so, we can enhance the efficiency of nursing workflows, reduce the burden on nurses, and potentially attract more individuals to the profession.

Moreover, improving the readiness of nursing students to adopt AI can lead to better patient outcomes by ensuring that future nurses are well equipped to utilize AI technologies in patient care. AI can assist in tasks such as patient monitoring, diagnostics, and personalized care plans, which can improve the quality of care provided. By understanding and addressing the specific attitudes and personality traits of Saudi Arabian nursing students, we can develop targeted educational strategies and interventions that foster a positive attitude toward AI, ultimately benefiting both nurses and patients.

To guide future strategies for promoting and applying AI in nursing, our findings suggest the need for targeted educational interventions that consider the diverse personality traits of nursing students. Training programs should emphasize the benefits and practical applications of AI in nursing to alleviate fears and resistance, particularly among those with high neuroticism and conscientiousness. Additionally, integrating AI-related content into nursing curricula can help build familiarity and competence, paving the way for smoother adoption and utilization of AI technologies in healthcare settings. These strategies can contribute to a more prepared and adaptable nursing workforce, capable of leveraging AI to improve patient outcomes and address the ongoing challenges in the healthcare sector.

### 4.3. Limitations

There are some limitations to the research. The initial multicenter, cross-sectional research methodology of this study made it harder to build a causal relationship between the identified factors and nurses' knowledge and attitudes toward artificial intelligence. A long-term study on the personality traits of nursing students is recommended in order to have a more comprehensive understanding of the students' traits throughout various points in time of their lives and in relation to various clinical and professional concerns. Second, there is a greater chance of source/response bias because self-reported measures were employed to collect the data simultaneously and using the same method: online. Future studies should consider using a variety of data sources, such as focused groups and individual interviews, in order to preserve objectivity.

Third, depending on their personality traits, some students showed a more positive attitude toward AI than others, while others showed a more tendency toward negative ones. The negative attitudes might be a result of a variety of complex aspects such as worries that artificial intelligence (AI) could outsmart humans, create moral/ethical dilemmas, or lead to loss of jobs. Therefore, it is crucial to take into account the social and cultural elements that can affect Saudi Arabians' attitudes toward artificial intelligence. Fourth, although the study was multicentered, it was limited to a specific sample of Saudi Arabian nursing students, which may have limited the findings' generalizability and application to other populations of nursing students or healthcare settings. It would be helpful to replicate this study with greater and more varied samples to confirm rigor and generalizability.

Another limitation is that while the study assessed nursing students' attitudes toward AI integration, it did not evaluate the possible actual incorporation of AI technology in their educational practices. Upcoming research could discover the real operation of AI tools by nursing students and evaluate its influence on their learning consequences and clinical training. Overall, these limitations highlight areas where improvements can be made in future studies to enhance our understanding of personality traits and attitudes toward AI among Saudi Arabian nursing students.

## 5. Conclusion

Artificial intelligence is developing swiftly and is already showing up in a number of health-related fields, including nursing. To properly integrate this technology into the nursing profession, it is therefore imperative to comprehend the elements that influence attitudes toward it. Overall, it appears that personality traits can influence individuals' attitudes toward AI, with certain traits being more closely related to positive or negative attitudes. Given the dearth of research on Saudi Arabian nursing students, the study's findings provide important insights into the personality traits and attitudes of Saudi Arabian nursing students toward artificial intelligence. The findings show that personality traits, except for extraversion, are often predictive of attitudes toward artificial intelligence whether positive or negative. The findings indicated that nursing students' positive attitudes toward artificial intelligence were strongly correlated with their openness to new experiences. Conversely, those whose personality traits scored highly on agreeableness and neuroticism were more likely to have negative than positive attitudes toward AI. Furthermore, conscientious nursing students have a negative attitude toward artificial intelligence.

## Figures and Tables

**Table 1:** Students' demographics and differences in attitude toward AI (*N* = 218).

Characteristics	Category	No.	%	Positive		Negative	
				*M* (SD)	*t*/*F* (*P*)	*M* (SD)	*t*/*F* (*P*)
Age (years)	<20	97	44.5	3.38 (0.78)	*t* = 0.19 (0.85)	2.39 (0.74)	*t* = 0.09 (0.92)
	≥20	121	55.5	3.36 (0.83)		2.38 (0.82)	

Gender	Male	40	18.3	3.21 (0.80)	*t* = −1.41 (0.17)	2.45 (0.88)	*t* = 0.52 (0.60)
	Female	178	81.7	3.40 (0.81)		2.37 (0.77)	

Marital status	Married	26	11.9	3.40 (0.81)	*t* = 0.23 (0.82)	2.32 (0.69)	*t* = −0.50 (0.62)
	Unmarried	192	88.1	3.36 (0.81)		2.39 (0.80)	

Educational level	1^st^ year	41	18.8	3.36 (0.75)	*F* = 1.31 (0.269)	2.21 (0.66)	*F* = 1.39 (0.237)
	2^nd^ year	36	16.5	3.62 (0.77)		2.31 (0.73)	
	3^rd^ year	40	18.4	3.33 (0.84)		2.61 (0.87)	
	4^th^ year	56	25.7	3.36 (0.83)		2.40 (0.83)	
	Intern	45	20.6	3.22 (0.80)		2.39 (0.79)	

*M*: mean; SD: standard deviation; *t*: independent-samples *t*-test; *F*: ANOVA test of variance; *P*: level of significance.

**Table 2:** Reliability, validity, and descriptive statistics (*N* = 218).

Variable	*α*	Factor loading	CR	AVE	MSV	Mean ± SD
Personality traits						
Extraversion	0.89	0.66–0.79	0.89	0.51	0.01	2.67 ± 0.81
Agreeableness	0.92	0.71–0.81	0.92	0.57	0.16	2.90 ± 0.77
Conscientiousness	0.90	0.68–0.75	0.90	0.51	0.13	3.18 ± 0.75
Neuroticism	0.90	0.63–0.78	0.90	0.53	0.16	2.18 ± 0.68
Openness	0.91	0.68–0.75	0.91	0.51	0.03	3.10 ± 0.75
Attitude toward AI						
Positive attitude toward AI	0.92	0.65–0.79	0.93	0.51	0.52	3.37 ± 0.81
Negative attitude toward AI	0.92	0.71–0.83	0.92	0.59	0.52	2.39 ± 0.79

AI: artificial intelligence; *α*: Cronbach's alpha; CR: composite reliability; AVE: average variance extracted; MSV: maximum shared variance; SD: standard deviation.

**Table 3:** Correlation analysis of personality traits and attitude toward AI (*N* = 218).

Personality traits	Attitude toward AI	
	Positive	Negative
Extraversion		
*r*	−0.002	−0.003
*p*	0.974	0.968
Agreeableness		
*r*	−0.153	0.488
*p*	0.024	<0.001
Conscientiousness		
*r*	−0.028	0.316
*p*	0.684	<0.001
Neuroticism		
*r*	−0.142	0.319
*p*	0.036	<0.001
Openness		
*r*	0.499	−0.118
*p*	<0.001	0.082

AI: artificial intelligence; *r*: Pearson's correlation; *p*: level of significance.

**Table 4:** Results of a multiple stepwise linear regression analysis predicting positive attitude toward AI of the studied nursing students (*N* = 218).

Predicators	*B*	SE (*B*)	*β*	*T*	*P* value	95% CI
Constant	2.623	0.260	—	10.075	<0.001	2.11–3.14
Openness	0.538	0.064	0.499	8.408	<0.001	0.412–0.664
Agreeableness	−0.186	0.066	−0.178	−2.822	0.005	−0.317–0.056
Neuroticism	−0.175	0.074	−0.149	−2.359	0.019	−0.322–0.029

*B*: unstandardized regression coefficient; SE: standard error; *β*: standardized regression coefficient; *T*: *T*-statistic; *P*: level of significance; 95% CI: 95% confidence interval; *R* (multiple correlation coefficient): 0.522; *R*^2^ (coefficient of determination): 0.272; Δ*R*^2^ (change in *R*^2^): 0.262; *F* (*F*-statistic): 26.695; *P* < 0.001.

**Table 5:** Results of a stepwise multiple linear regression analysis predicting negative attitude toward AI of the studied nursing students (*N* = 218).

Predicators	*B*	SE (*B*)	*β*	*T*	*P* value	95% CI
Constant	0.302	0.245	—	1.231	0.220	−0.181–0.785
Agreeableness	0.381	0.067	0.372	5.701	<0.001	0.249–0.512
Conscientiousness	0.181	0.064	0.172	2.816	0.005	0.054–0.308
Neuroticism	0.185	0.072	0.160	2.581	0.011	0.044–0.326

*B*: unstandardized regression coefficient; SE: standard error; *β*: standardized regression coefficient; *T*: *T*-statistic; *P*: level of significance; 95% CI: 95% confidence interval; *R* (multiple correlation coefficient): 0.536; *R*^2^ (coefficient of determination): 0.287; Δ*R*^2^ (change in *R*^2^): 0.277; *F* (*F*-statistic): 28.719; *P* < 0.001.

## Data Availability

The data used to support the findings of this study are available from the corresponding author upon request.
